# Repulsive guidance molecule-a regulates neutrophil-associated Th17 responses in CNS autoimmunity

**DOI:** 10.3389/fimmu.2026.1941039

**Published:** 2026-08-19

**Authors:** Weizhe Zhen, Xiaoyi Yang, Jinsong Jiao, Lei Cui, Miaoxin Yu, Qing Sun, Dianjia Gao, Dantao Peng, Ming Jin, Lai Wei, Weihe Zhang

**Affiliations:** 1 Graduate School, Capital Medical University, Department of Neurology, China-Japan Friendship Hospital, Beijing, China; 2 Graduate School, Beijing University of Chinese Medicine, Beijing, China; 3 Department of Neurology, China-Japan Friendship Hospital, Beijing, China; 4 Graduate School, Chinese Academy of Medical Sciences and Peking Union Medical College, Beijing, China; 5 Department of Ophthalmology, China-Japan Friendship Hospital, Beijing, China; 6 Guangdong Provincial Key Laboratory of Allergy and Clinical Immunology, Institute of Ophthalmology, The Second Affiliated Hospital, Guangzhou Medical University, Guangzhou, China

**Keywords:** BMP4-SMAD pathway, multiple sclerosis, neuroinflammation, neutrophil-Th17 axis, RGMa

## Abstract

**Background and aim:**

Repulsive guidance molecule-A (RGMa) is well-known for its roles in T-cell-mediated neuroinflammation and CNS repair. Innate-adaptive immune interactions, particularly between neutrophils and Th17 cells, are emerging as critical drivers of neuroinflammatory pathology in multiple sclerosis (MS). Whether RGMa regulates these neutrophil-associated Th17 responses remains largely unknown. Elucidating this mechanism may reveal novel therapeutic targets for MS.

**Methods:**

Serum levels of RGMa, BMP4, neutrophil elastase (NE), IL-17A, and inflammatory markers were measured in 10 relapsing-remitting MS patients during acute relapse and remission phases, and in 10 healthy controls. *In vitro* transwell co-cultures using primary human neutrophils and CD3^+^ T cells were employed to evaluate the effects of RGMa monoclonal antibody (RGMa-mAb) treatment on neutrophil activation, Th17-associated responses, and T-cell migration. In parallel, experimental autoimmune encephalomyelitis (EAE) mice were treated with RGMa-mAb to assess neuroinflammation and BMP4-SMAD-related signaling changes *in vivo*.

**Results:**

Serum RGMa and NE were elevated in acute-phase MS patients versus both remission and healthy controls (*p* < 0.05), whereas IL-17A was elevated versus healthy controls (*p* < 0.05). Correlation analyses showed that RGMa levels were positively associated with the neutrophil activation marker NE (*r* = 0.71), while serum Ly6G6D levels were correlated with IL-17A levels (*r* = 0.66), suggesting a potential link between neutrophil-associated inflammatory responses and Th17 activation during active disease. *In vitro*, RGMa-mAb treatment attenuated PMA-induced neutrophil activation, reduced Th17-associated inflammatory responses, and suppressed T-cell migration in the co-culture system. In EAE mice, RGMa-mAb ameliorated neurological deficits, decreased neutrophil- and Th17-associated inflammatory markers, and modulated BMP4-SMAD signaling.

**Conclusion:**

RGMa contributes to neutrophil-associated Th17 inflammatory responses in CNS autoimmunity, potentially in association with BMP4-SMAD signaling. Targeting RGMa may therefore represent a potential therapeutic strategy for MS-associated neuroinflammation that warrants further investigation.

## 1 Introduction

Multiple sclerosis (MS) is a chronic immune-mediated disorder of the central nervous system (CNS) and a leading cause of non-traumatic neurological disability in young adults worldwide (1–5). Its pathogenesis is characterized by the infiltration of both innate and adaptive immune cells, including autoreactive T and B cells and neutrophils, which contribute to myelin destruction and axonal injury (3). Recent studies highlight the importance of immune-cell-associated signaling pathways in modulating neuroinflammatory responses in preclinical MS models (6, 7).

Among these immune cells, T helper 17 (Th17) cells are considered major contributors in disease development and progression by secreting pro-inflammatory cytokines such as interleukin-17 (IL-17), which amplifies CNS inflammation (8–11). Several signaling pathways, including MAPK signaling, have been implicated in regulating Th17 differentiation in the context of experimental autoimmune encephalomyelitis (EAE) (12, 13), further supporting the role of immune signaling in Th17-driven neuroinflammation. Concurrently, neutrophils, once considered secondary responders, are now recognized as active contributors to neuroinflammation, instigating tissue injury through the release of soluble mediators and neutrophil extracellular traps (NETs) (14, 15). Importantly, Th17 cells and neutrophils interact through a self-reinforcing “neutrophil–Th17 axis”: neutrophil-derived components promote Th17 cell differentiation, while Th17-derived IL-17 reciprocally activates neutrophils, forming a positive feedback loop that sustains inflammation and tissue injury (16, 17). This feedback loop has increasingly been implicated in acute immunopathology in MS and other autoimmune disorders (16, 18).

Repulsive guidance molecule A (RGMa), initially characterized in axonal guidance, has recently been identified as a potent immunomodulator. In EAE, RGMa expression is elevated in the inflamed CNS, where it promotes T cell responses by enhancing T cell adhesion and contributes to the pathogenic Th17 cell responses (19). Moreover, RGMa signaling has been shown to activate macrophages and mediate macrophage-dependent neutrophil recruitment, highlighting its role within the innate immune network (20). Notably, previous studies suggest that RGMa may regulate immune cell activation via bone morphogenetic protein (BMP) signaling pathways (21, 22).

Although both RGMa signaling has been implicated in T-cell-mediated CNS autoimmunity and neutrophil-Th17 inflammatory responses have also been associated with neuroinflammation, the potential relationship between these processes remains poorly understood. Given the emerging role of RGMa in regulating multiple immune-cell-associated inflammatory responses in CNS autoimmunity, we hypothesized that RGMa may contribute to neuroinflammation by modulating neutrophil-associated Th17 immune interactions, thereby linking innate and adaptive immune compartments. This perspective highlights the importance of targeting pathogenic immune-cell interactions rather than isolated inflammatory mediators. To address this question, we utilized clinical samples, *in vitro* co-culture systems, and *in vivo* EAE models to investigate the role of RGMa in neutrophil-associated Th17 responses and the associated signaling pathways in CNS autoimmunity.

## 2 Materials and methods

### 2.1 Participants and sample collection

A total of 20 individuals were enrolled in this study, including 10 patients with relapsing-remitting multiple sclerosis (RRMS) and 10 age- and sex-matched healthy controls (HCs). RRMS patients were diagnosed according to the 2017 revised McDonald criteria (23) and blood samples were collected during an acute relapse (pre-treatment) and during remission (≥3 months post-relapse). Peripheral blood (4 mL) was centrifuged at 1,500 × g for 10 min at 4 °C to isolate serum, which was aliquoted and stored at −80 °C until analysis. All participants were recruited from the Department of Neurology, China–Japan Friendship Hospital. This study was approved by the Institutional Ethics Committee (2025-KY-333), and written informed consent was obtained from all participants.

### 2.2 Isolation and preparation of primary human neutrophils

Peripheral blood from healthy subjects was subjected to density gradient centrifugation using Ficoll-Paque Plus (Cytiva) followed by negative selection with human neutrophil isolation kit (Miltenyi Biotec, #130-104-434) according to the manufacturer’s instructions. Erythrocytes were lysed using ammonium chloride buffer (STEMCELL Technologies, #07800). The purity of the isolated neutrophils was consistently above 95%, and the cell viability was greater than 98%, as determined by flow cytometry and trypan blue exclusion, respectively.

### 2.3 Isolation of primary human T lymphocytes

Peripheral blood mononuclear cells (PBMCs) were isolated from whole blood of healthy subjects by density gradient centrifugation with Lymphopaque™ (Cytiva, #17-829-02). Primary human CD3^+^ T lymphocytes were then purified from PBMCs using the Human Pan-T Cell Isolation Kit (Miltenyi Biotec, #130-096-535) following the manufacturer’s instructions. The purity of the isolated CD3^+^ T lymphocytes was consistently above 95%, and the cell viability was greater than 98%, as confirmed by flow cytometry and trypan blue exclusion, respectively.

### 2.4 Co-culture of neutrophils and T cells

Purified human neutrophils were divided into three groups: (i) Control group, in which neutrophils received no stimulation; (ii) PMA group, in which neutrophils were treated with 100 nM PMA for 4 h; and (iii) RGMa-mAb group, in which neutrophils were treated with 100 nM PMA together with 1 μg/mL RGMa-mAb for 4 h. Following treatment, neutrophils were seeded into the lower chamber of a transwell system (LabSelect, #14322) at 5 × 10^5^ cells per well. CD3^+^ T cells were added to the upper chamber at the same density and co-cultured for an additional 24 h using a 3-μm pore-size membrane, which prevents direct cell-cell contact while allowing diffusion of soluble mediators. Detailed information regarding antibodies and experimental reagents is provided in **Supplementary Table 1**.

### 2.5 Flow cytometry analysis

Cells were harvested after co-culture and washed with PBS. To exclude dead cells and debris, cells were initially gated based on forward scatter (FSC) and side scatter (SSC) characteristics. For surface staining, cells were incubated with fluorescently conjugated antibodies against CD11b (PE), Ly-6G/Ly-6C (FITC), and CD4 (FITC) in the dark at 4 °C for 30 min. For intracellular IL-17A (PE) staining, cells were fixed and permeabilized using a commercial fixation/permeabilization buffer (e.g., eBioscience Foxp3/Transcription Factor Staining Buffer Set) according to the manufacturer’s protocol. Briefly, cells were fixed for 30 min at 4 °C, then permeabilized and stained with anti-IL-17A antibody in the provided permeabilization buffer. All antibody staining was performed in the dark at 4 °C for 30 min. After washing, cells were analyzed on a CytoFLEX flow cytometer (Beckman Coulter). Isotype and unstained controls were included to validate gating and staining specificity. For the co-culture experiments, three independent experiments were performed using neutrophils and T cells isolated from the same healthy donor to assess experimental reproducibility. Representative gating strategies for neutrophil (CD11b^+^Ly-6G/Ly-6C^+^) and Th17-cell (CD4^+^IL-17A^+^) populations are provided in **Supplementary Figure 1**. Data analysis was performed using FlowJo software (BD Biosciences).

### 2.6 T cell migration assay

T cell migration was assessed using a transwell system (LabSelect, #14322) with a 3-μm pore-size membrane following co-culture. After a 24-hour incubation at 37 °C with 5% CO_2_, T cells that had migrated to the lower membrane surface were fixed, stained with crystal violet, and manually counted under a light microscope.

### 2.7 EAE induction, treatment and neurological assessment

EAE was induced in 8- to 10-week-old female C57BL/6 mice by subcutaneous injection of an emulsion containing 150 μg MOG_35_–_55_ peptide in Complete Freund’s Adjuvant (CFA, 1 mg/mL) supplemented with 4 mg/mL Mycobacterium tuberculosis H37 Ra (final concentration), followed by intravenous administration of 200 ng pertussis toxin (PTX) on day 0 and day 2 post-induction. Mice were randomly assigned to three groups (n=5 per group) using a computer-generated randomization sequence: Control, EAE, and EAE + RGMa-mAb (RGMa-mAb). For histological and immunofluorescence analyses, three of the five animals per group were randomly selected using a random number table. This sample size is consistent with group sizes commonly used in exploratory EAE studies. The treatment group received RGMa-mAb (10 mg/kg) via tail vein injection once weekly for two weeks, beginning during the early stage following EAE induction before overt neurological deficits developed. Disease progression was monitored daily for 14 days. Neurological function was evaluated at baseline (day 0, immediately before treatment initiation), day 7, and day 14. Neurological scoring was performed in a blinded manner according to the following criteria: 0: no symptoms; 1: complete tail atony; 2: hindlimb weakness; 3: hindlimb paralysis; 4: complete hindlimb paralysis and forelimb weakness; 5: moribund or death. All animal procedures were approved by the Animal Care and Use Committee of Beijing Shenrui Biotechnology Co., Ltd. (Approval No. SR20250916). Detailed information regarding the antibodies and key experimental reagents used in this study, including catalog numbers and specifications, is provided in **Supplementary Table 1**.

### 2.8 Enzyme-linked immunosorbent assay

Human serum levels of RGMa, BMP4, NE, IL-17A, and IFN-γ were measured using commercially available ELISA kits (Ruixin Biotechnology, China), while human Ly6G6D levels were specifically quantified using a human Ly6G6D ELISA kit (Jingmei Biotechnology, China). In addition, NE, CD66, IFN-γ, MPO, and IL-17A levels in mouse tissues and cell culture supernatants were measured using ELISA kits (Ruixin Biotechnology, China) according to the manufacturers’ protocols. All assays were performed in duplicate, and absorbance was measured at 450 nm using a microplate reader. Detailed information on each ELISA kit, including catalog numbers, species reactivity, and specifications, is provided in **Supplementary Table 1**.

### 2.9 Histological staining

Brain and spinal cord tissues were fixed in 10% formalin, embedded in paraffin, and sectioned at 5 μm thickness. Tissue sections were then stained with hematoxylin and eosin (H&E) for histological evaluation of inflammatory infiltration and tissue morphology using an Olympus BX53 microscope.

### 2.10 Immunofluorescence and TUNEL staining

For immunofluorescence staining, formalin-fixed paraffin-embedded brain and spinal cord sections were deparaffinized, rehydrated, and subjected to antigen retrieval using citrate buffer (pH 6.0) at 95 °C for 15 min. Sections were then blocked with 5% bovine serum albumin (BSA) for 1 h at room temperature and incubated overnight at 4 °C with specific primary antibodies, followed by fluorophore-conjugated secondary antibodies for 1 h at room temperature in the dark. Nuclei were counterstained with DAPI. Apoptotic cells were detected using a TUNEL assay kit according to the manufacturer’s instructions. All stained sections were imaged using a fluorescence microscope under identical acquisition settings.

### 2.11 Quantitative real-time PCR

Total RNA was extracted from cells or tissues using TRIzol reagent (Thermo Fisher Scientific), and cDNA was synthesized from 1 µg RNA using the PrimeScript RT Master Mix (Takara). Quantitative PCR was performed on a Bio-Rad CFX96 system using SYBR Green Premix Pro Taq HS (Bio-Rad) with the following protocol: 95 °C for 30 s, followed by 40 cycles of 95 °C for 5 s and 60 °C for 30 s. A melting curve analysis was included to confirm amplification specificity. Relative gene expression was quantified using the comparative 2^^–ΔΔCt^ method, normalized to GAPDH expression and expressed relative to the corresponding control group. All reactions were performed in technical triplicates. The primer sequences for all target genes (*RGMa, BMP4, MPO, SMAD1, SMAD4, SMAD5, SMAD8*) and the reference gene (*GAPDH*) are listed in **Supplementary Tables 2** and **3**.

### 2.12 Western blotting

Total protein was extracted from neutrophils and spinal cord tissues using RIPA lysis buffer (Beyotime) supplemented with protease and phosphatase inhibitors. Protein concentrations were determined with a BCA assay kit (Beyotime). Equal amounts of protein (30µg per lane) were separated by SDS-PAGE and transferred to PVDF membranes. After blocking with 5% non-fat milk for 1 h, membranes were incubated overnight at 4 °C with primary antibodies against targets including RGMa, NE, MPO, BMP4, IL-17A, SMAD1/4/5/8, and GAPDH (loading control), followed by incubation with HRP-conjugated secondary antibodies for 1 h at room temperature. Protein bands were visualized using an enhanced chemiluminescence (ECL) detection kit (Thermo Fisher Scientific) and quantified with ImageJ software, normalized to GAPDH. Detailed information on all primary and secondary antibodies, including catalog numbers, host species, dilution ratios, and applications, and the expected molecular weights of target proteins, is provided in **Supplementary Table 1**.

### 2.13 Statistical analysis

Data are presented as mean ± standard deviation (SD) from at least three independent experiments. All statistical analyses were performed using SPSS (version 25.0) and GraphPad Prism (version 8.0). Differences between two groups were assessed by an unpaired two-tailed Student’s t-test, while comparisons among multiple groups were performed using one-way ANOVA followed by Tukey’s *post hoc* test. Correlation analyses were conducted using Spearman’s rank correlation. *P*-value < 0.05 was considered statistically significant.

## 3 Results

### 3.1 Patients with acute-phase MS exhibited elevated serum levels of RGMa, IL-17A, and NE

A total of 10 patients with RRMS were enrolled in this study (7 females and 3 males, aged 24–45 years). Six patients presented with their first clinical episode, while four had a history of more than one relapse prior to enrollment. Clinical manifestations at onset included cerebral lesions (n = 4), myelitis (n = 3), brainstem lesions (n = 2), and optic neuritis (n = 1). The nadir Expanded Disability Status Scale (EDSS) scores during the acute phase ranged from 2 to 5. Cerebrospinal fluid oligoclonal bands were positive in all patients. No patients had comorbid autoimmune disorders or uncontrolled infectious diseases. All patients received high-dose intravenous methylprednisolone pulse therapy during the acute phase. Patients in remission were maintained on disease-modifying therapies (DMTs), including ofatumumab (n = 6), siponimod (n = 2), or teriflunomide (n = 2). At the 3-month follow-up, EDSS scores improved to 0-1. Healthy controls matched for age and sex were also included.

**Figure 1:**
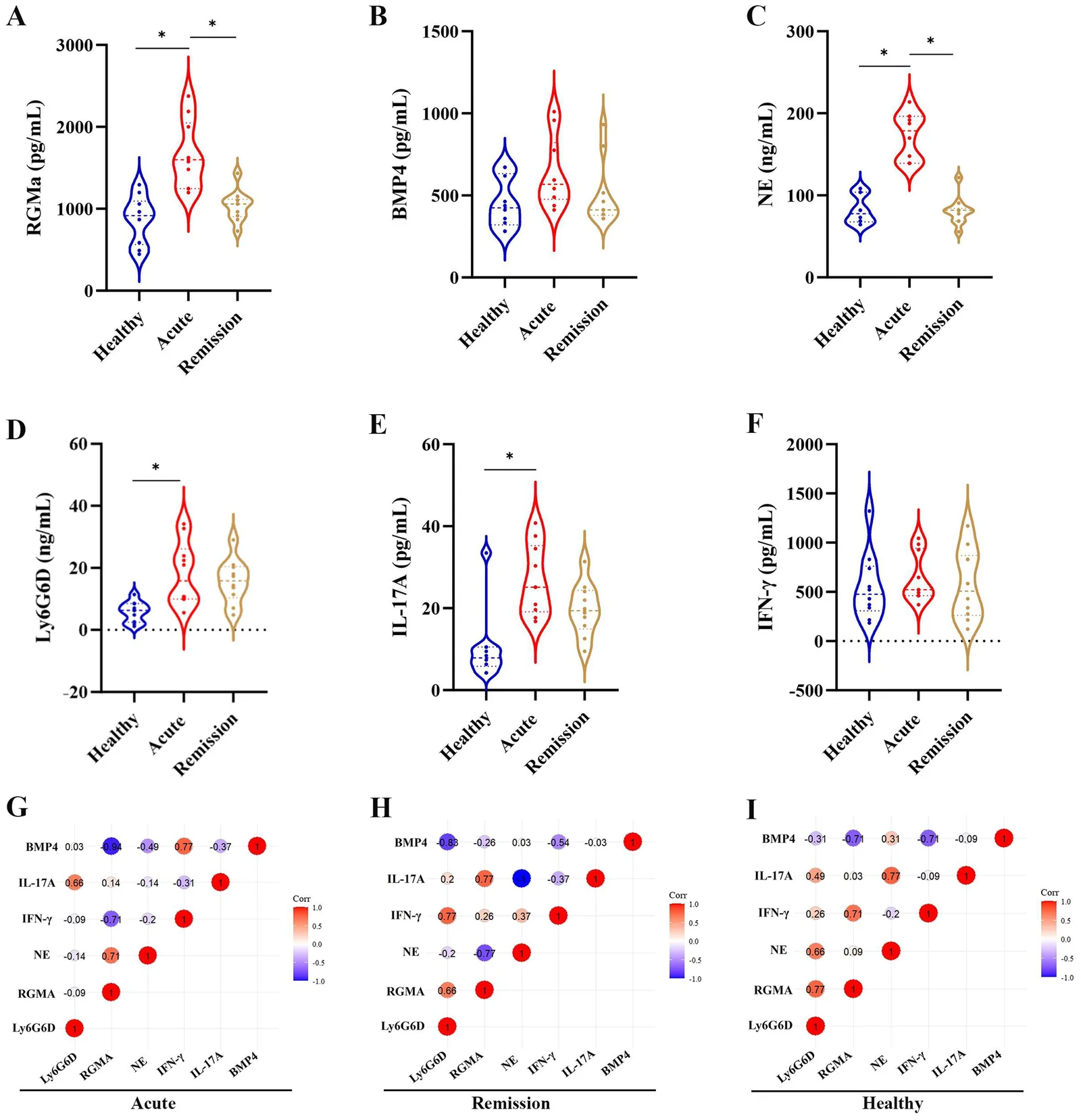
Group comparisons and correlation analysis of RGMa-related inflammatory markers in acute MS, remission, and healthy controls. **(A–F)** Violin plot comparisons of RGMa, BMP4, NE, Ly6G6D, IL-17A, and IFN-γ among the three groups. Statistical significance is indicated as follows: *p < 0.05. **(G–I)** Correlation heatmaps among six immunological markers in acute-phase MS patients, remission-phase MS patients, and healthy controls. Correlation coefficients (r) were calculated using Spearman’s rank correlation analysis. Sample sizes: acute-phase MS and remission-phase groups consisted of paired samples from the same 10 patients, while the healthy control group included 10 independent donors. BMP4, bone morphogenetic protein 4; IFN-γ, interferon-gamma; IL-17A, interleukin-17A; Ly6G6D, lymphocyte antigen 6 family member G6D; NE, neutrophil elastase; RGMa, repulsive guidance molecule-A.

As shown in **Figures 1A–F**, serum concentrations of RGMa, NE, and IL-17A were significantly higher in acute-phase MS patients compared with healthy controls (*p* < 0.05 for all comparisons). In addition, RGMa and NE levels were significantly higher in acute-phase patients compared with remission-phase patients (*p* < 0.05 for all comparisons).

Correlation analysis revealed a distinct disease-associated pattern linking RGMa, neutrophil activation, and the Th17 pathway (**Figures 1G–I**). Notably, this inflammatory correlation profile was more apparent in acute-phase MS patients. Within this group, RGMa levels were strongly associated with NE (*r* = 0.71), a marker of neutrophil activation, whereas serum Ly6G6D protein levels showed a significant correlation with IL-17A (*r* = 0.66). In contrast, RGMa did not significantly correlate with Ly6G6D during active disease (r = -0.09), suggesting that RGMa may be more closely linked to neutrophil activation rather than neutrophil abundance in acute inflammation. Together, these findings support a potential association between RGMa signaling, neutrophil activation, and Th17-associated inflammatory responses during active disease.

During remission, the acute-phase correlation pattern between RGMa, neutrophil activation, and Th17-associated inflammation was no longer preserved. Specifically, the correlation between RGMa and NE was absent, whereas RGMa showed a moderate association with Ly6G6D, which may reflect residual neutrophil abundance rather than activation status during disease remission. In addition, the relationship between NE and IL-17A differed from that observed during acute disease. No significant coordinated correlations among these mediators were found in healthy controls (*p* > 0.05 for all comparisons).

### 3.2 RGMa-mAb treatment attenuates Th17 polarization and T-cell migration induced by PMA-activated neutrophils

**Figure 2:**
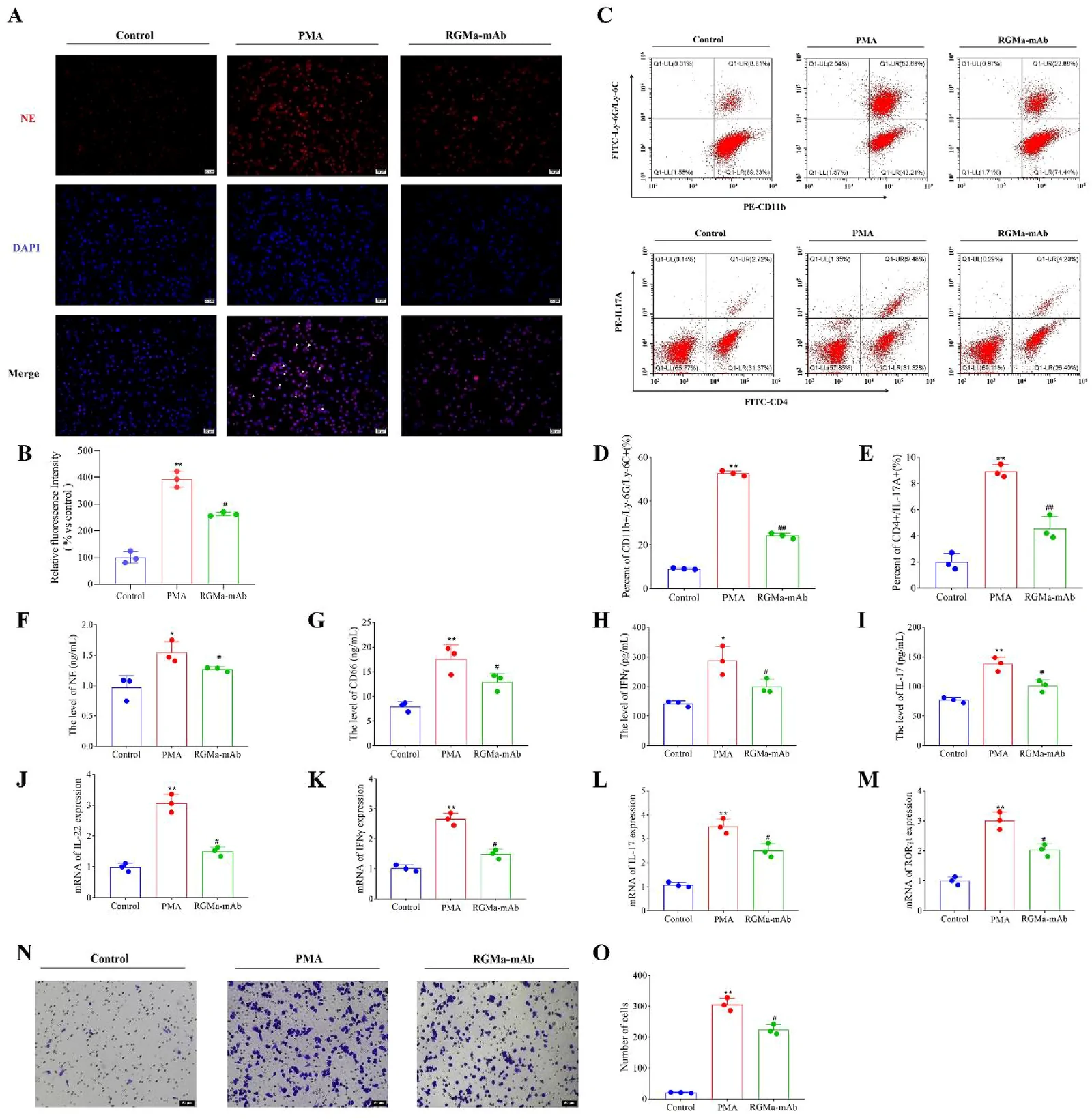
RGMa-mAb treatment attenuates neutrophil activation and suppresses neutrophil-associated Th17 inflammatory responses *in vitro*. **(A, B)** Representative immunofluorescence images and quantitative analysis of NE fluorescence intensity in neutrophils under Control, PMA, and RGMa-mAb conditions. NE is shown in red, and nuclei are counterstained with DAPI (blue). **(C–E)** Representative flow cytometry plots **(C)** and quantitative analysis of CD11b^+^Ly-6G/Ly-6C^+^ neutrophils **(D)** and CD4^+^IL-17A^+^ Th17 cells **(E)** after neutrophil-T cell co-culture under different treatment conditions. **(F–I)** ELISA analysis of NE, CD66, IFN-γ, and IL-17A levels in culture supernatants after neutrophil-T cell co-culture under different treatment conditions. **(J–M)** RT-qPCR analysis of RORγt, IL-17A, IL-22, and IFN-γ mRNA expression levels in T cells following co-culture with neutrophils under different treatment conditions. **(N, O)** Representative images and quantitative analysis of the T-cell transwell migration assay showing migrated T cells stained with crystal violet after co-culture with neutrophils under different treatment conditions. Neutrophils were divided into three groups: Control group (unstimulated neutrophils), PMA group (100 nM PMA stimulation for 4 h), and RGMa-mAb group (100 nM PMA plus 1 μg/mL RGMa-mAb for 4 h). Neutrophils were pretreated with PMA or PMA plus RGMa-mAb before co-culture with CD3^+^ T cells. Neutrophils were then seeded in the lower chamber, while CD3^+^ T cells were placed in the upper chamber of a 3-μm transwell system to prevent direct cell-cell contact while allowing diffusion of soluble mediators. Data are presented as mean ± SD from three independent experiments performed using cells from the same healthy donor (n = 3 independent experiments per group). Quantitative immunofluorescence and migration analyses were performed using three randomly selected microscopic fields per sample. Scale bars are indicated in each panel. Statistical analyses were performed using one-way ANOVA followed by multiple-comparison testing. *p < 0.05 and **p < 0.01 versus the Control group; #p < 0.05 and ##p < 0.01 versus the PMA group. DAPI, 4’,6-diamidino-2-phenylindole; ELISA, enzyme-linked immunosorbent assay; IFN-γ, interferon-gamma; IL-17A, interleukin-17A; IL-22, interleukin-22; NE, neutrophil elastase; RGMa-mAb, repulsive guidance molecule-A monoclonal antibody; RORγt, retinoic acid receptor-related orphan receptor gamma t; RT-qPCR, reverse transcription polymerase chain reaction.

To investigate the influence of neutrophil activation on Th17-associated inflammatory responses, we established a transwell co-culture system using isolated neutrophils and CD3^+^ T cells. Immunofluorescence analysis showed that PMA stimulation significantly elevated the expression of NE (*p* = 0.0081), a marker associated with neutrophil activation, which was effectively suppressed by RGMa-mAb treatment (*p* = 0.0342; **Figures 2A, B**).

Neutrophil activation and Th17 differentiation were further evaluated by flow cytometry. PMA stimulation markedly increased the percentage of CD11b^+^Ly-6G/Ly-6C^+^ neutrophils compared with the control (*p* = 0.0026). This increase was significantly attenuated by RGMa-mAb treatment (*p* = 0.0049) (**Figures 2C–E**). Correspondingly, PMA-activated neutrophils were associated with increased frequencies of CD4^+^IL-17A^+^ T cells, as shown by a higher frequency of CD4^+^IL-17A^+^ T cells relative to the control (*p* = 0.0057). This increase in Th17-associated inflammatory responses was also substantially suppressed by RGMa-mAb treatment (*p* = 0.0086). After co-culture with PMA-activated neutrophils, the levels of NE, CD66, IFN-γ and IL-17A in the supernatant were significantly elevated (*p* < 0.05 for all comparisons), as determined by ELISA (*p* < 0.05 for all comparisons; **Figures 2F–I**), and these increases were significantly attenuated by RGMa-mAb treatment. At the transcriptional level, PMA-induced neutrophil activation led to significant upregulation of *RORγt*, *IL-17A*, *IL-22*, and *IFN-γ* in T cells (*p* < 0.05 for all comparisons). RGMa-mAb treatment significantly suppressed the mRNA expression of these pro-inflammatory mediators (*p* < 0.05 for all comparisons; **Figures 2J–M**).

Finally, we evaluated the chemotactic influence of activated neutrophils on T cells. Compared with the control, PMA-activated neutrophils induced substantial T-cell migration (*p* = 0.0013), which was strongly suppressed by RGMa-mAb treatment (*p* = 0.0442) (**Figures 2N, O**).

Importantly, RGMa-mAb intervention was restricted to the neutrophil compartment before co-culture with CD3^+^ T cells. Because the transwell system prevented direct cell-cell contact while allowing diffusion of soluble mediators, the observed changes in T-cell activation and IL-17A production likely reflect indirect effects mediated by neutrophil-derived inflammatory signals. Collectively, these findings suggest that RGMa is associated with neutrophil–Th17 inflammatory interactions *in vitro*.

### 3.3 RGMa-mAb alleviates neurological deficits and CNS pathology in EAE mice

**Figure 3:**
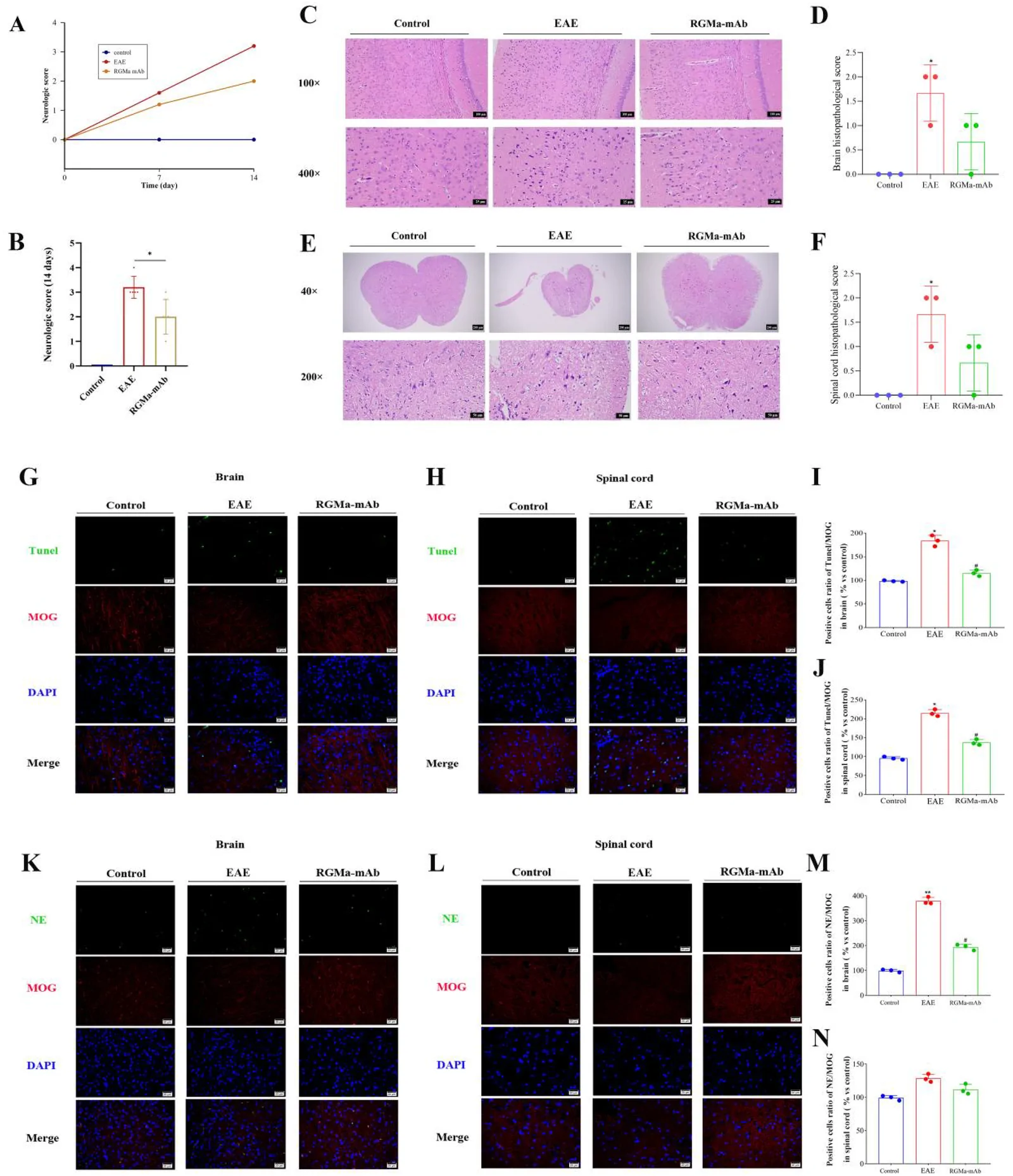
RGMa-mAb treatment alleviates neurological deficits, inflammatory pathology, MOG-associated apoptotic injury, and neutrophil infiltration in EAE mice. **(A, B)** Neurological function scores of Control, EAE, and RGMa-mAb-treated mice during the 14-day observation period and quantitative analysis at the experimental endpoint. Control mice showed no clinical signs of EAE, with scores remaining at 0 throughout the observation period. **(C, D)** Representative H&E staining images of brain tissue **(C)** and corresponding quantitative inflammatory infiltration scores **(D)**. **(E, F)** Representative H&E staining images of spinal cord tissue **(E)** and corresponding quantitative inflammatory infiltration scores **(F)**. Scale bars are indicated in each panel. **(G–J)** Representative TUNEL and MOG immunofluorescence co-staining images and quantitative analysis of apoptotic signals associated with MOG-positive regions in brain and spinal cord tissues. TUNEL-positive cells are shown in green, MOG-positive regions in red, and nuclei are counterstained with DAPI (blue). **(K–N)** Representative immunofluorescence images and quantitative analysis of neutrophil elastase (NE) and MOG staining in brain and spinal cord tissues from different treatment groups. NE is shown in green, MOG in red, and nuclei are counterstained with DAPI (blue). Mice were divided into three groups: Control, EAE, and RGMa-mAb treatment groups. RGMa-mAb was administered via tail vein injection (10 mg/kg) once weekly for two weeks following EAE induction. Data are presented as mean ± SD. Neurological scoring analyses included five mice per group (n = 5), while representative histological and immunofluorescence analyses were obtained from three randomly selected mice per group. Scale bars are indicated in each panel. Quantitative histological and immunofluorescence analyses were performed using three randomly selected microscopic fields from three tissue sections per animal. Statistical significance is indicated as follows: *p < 0.05 and **p < 0.01 versus the Control group; #p < 0.05 and ##p < 0.01 versus the EAE group. DAPI, 4’,6-diamidino-2-phenylindole; EAE, experimental autoimmune encephalomyelitis; H&E, hematoxylin and eosin; MOG, myelin oligodendrocyte glycoprotein; NE, neutrophil elastase; RGMa-mAb, repulsive guidance molecule-A monoclonal antibody; TUNEL, terminal deoxynucleotidyl transferase dUTP nick end labeling.

The therapeutic effect of RGMa-mAb on neurological function was assessed in EAE mice. As shown in **Figures 3A, B**, RGMa-mAb treatment significantly improved neurological function scores compared with the untreated EAE group after 14 days of treatment (*p* = 0.0412). Histopathological examination revealed marked edema and inflammatory cell infiltration in the brain and spinal cord tissues of EAE mice. Quantitative histopathological scoring further confirmed significantly increased inflammatory infiltration in EAE mice compared with controls (*p* < 0.05), whereas RGMa-mAb treatment attenuated these pathological changes and reduced inflammatory infiltration scores in both CNS regions (**Figures 3C–F**).

### 3.4 RGMa inhibition attenuates MOG-associated apoptotic injury and neutrophil infiltration in EAE mice

Given the importance of myelin and oligodendrocyte-associated pathology in EAE, we next evaluated apoptotic injury within MOG-positive regions. TUNEL and MOG immunofluorescence staining revealed increased apoptotic signals associated with MOG-positive regions in EAE mice. RGMa-mAb treatment significantly reduced these pathological changes (*p* < 0.05; **Figures 3G–J**), suggesting RGMa inhibition attenuates MOG-associated apoptotic injury.

Concurrently, immunofluorescence staining for NE showed increased neutrophil-associated inflammatory infiltration in the CNS tissue of EAE mice compared with controls (*p* = 0.0013). RGMa-mAb treatment attenuated NE-associated inflammatory signals and reduced neutrophil accumulation within MOG-positive regions (*p* = 0.0169; **Figures 3K–N**). These findings suggest that RGMa inhibition may contribute to attenuation of neutrophil-associated tissue injury in EAE lesions.

### 3.5 RGMa is associated with neutrophil-Th17 inflammatory responses in the CNS of EAE mice

To investigate whether RGMa is associated with neutrophil-Th17 inflammatory responses within the inflamed CNS, we quantified the levels of neutrophil-associated inflammatory markers (CD66 and MPO) and Th17-associated cytokines (IFN-γ and IL-17A) in the brain and spinal cord tissues of EAE mice by ELISA.

**Figure 4:**
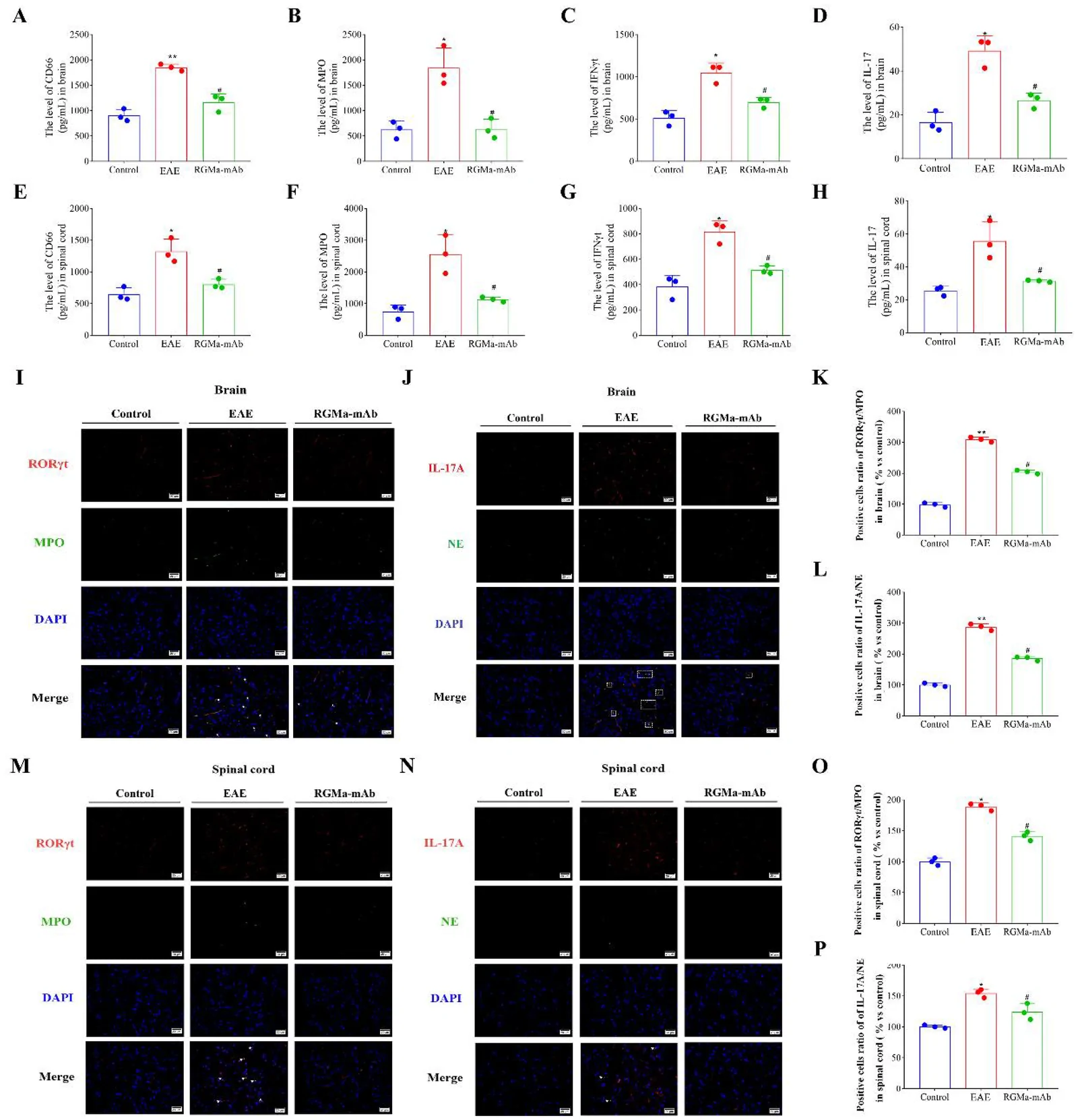
RGMa-mAb disrupts neutrophil-Th17 inflammatory interactions in CNS tissues of EAE mice. **(A–H)** Quantitative analysis of inflammatory mediators in brain [**(A, B)**, neutrophil-associated inflammatory markers CD66, MPO; **(C, D)**, Th17-associated cytokines IFN-γ, IL-17A] and spinal cord [**(E, F)**, neutrophil-associated inflammatory markers CD66, MPO; **(G, H)**, Th17-associated cytokines IFN-γ, IL-17A] tissues. ELISA measurements show significantly elevated levels in EAE mice compared with Control mice, which were effectively attenuated by RGMa-mAb treatment. **(I–P)** Spatial analysis of neutrophil-Th17 interactions by dual immunofluorescence staining. Brain tissue: MPO^+^ neutrophils with RORγt^+^ Th17 cells **(I)** and NE^+^ neutrophils with IL-17A^+^ Th17 cells **(J)**; quantitative analysis is shown in **(K, L)**. Spinal cord tissue: MPO^+^ neutrophils with RORγt^+^ Th17 cells **(M)** and NE^+^ neutrophils with IL-17A^+^ Th17 cells **(N)**; quantitative analysis is shown in **(O, P)**. RGMa-mAb treatment altered the spatial association between neutrophils and Th17 cells in both CNS regions, consistent with attenuation of neutrophil-associated Th17 inflammatory interactions. DAPI (blue) marks nuclei. All images and quantifications represent three independent experiments (n = 3 animals per group). Quantitative immunofluorescence analyses were performed using three tissue sections per animal and three randomly selected microscopic fields per section. Scale bars are indicated in each panel. Statistical analyses were performed using one-way ANOVA followed by multiple-comparison testing. Differences between EAE and Control groups are indicated by *p < 0.05 and **p < 0.01; differences between RGMa-mAb and EAE groups are indicated by #p < 0.05 and ##p < 0.01. DAPI, 4’,6-diamidino-2-phenylindole; EAE, experimental autoimmune encephalomyelitis; ELISA, enzyme-linked immunosorbent assay; IFN-γ, interferon-gamma; IL-17, interleukin-17; RGMa-mAb, repulsive guidance molecule-A monoclonal antibody; NE, neutrophil elastase; RGMa-mAb, repulsive guidance molecule-A monoclonal antibody; RORγt, retinoic acid receptor-related orphan receptor gamma t.

The results demonstrated that the levels of CD66, MPO, IFN-γ, and IL-17A were significantly elevated in EAE mice compared with controls (*p* < 0.05 for all comparisons), and RGMa-mAb treatment attenuated this elevation (*p* < 0.05 for all comparisons, **Figures 4A–H**). Dual immunofluorescence staining showed spatial proximity between neutrophil markers (MPO/NE) and Th17-associated markers (RORγt/IL-17A) within CNS lesions. RGMa inhibition reduced the intensity of these signals (*p* < 0.05) and was associated with decreased spatial overlap between these marker populations (**Figures 4I–P**). These findings are consistent with a spatial association or overlap between neutrophil-associated and Th17-associated markers.

### 3.6 RGMa-associated BMP4-SMAD signaling alterations in neutrophils during EAE

**Figure 5:**
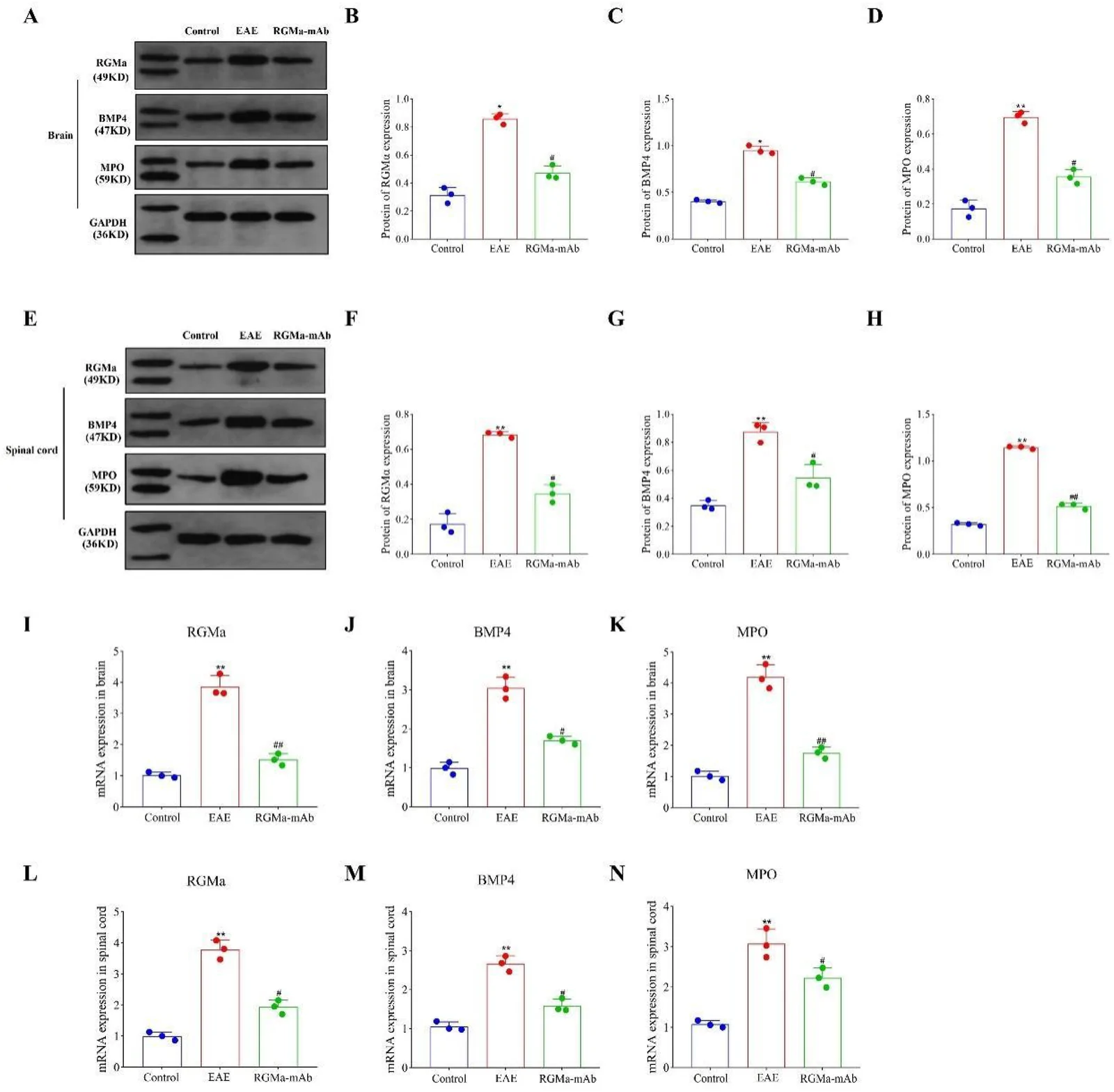
RGMa-mAb intervention reduces RGMa, BMP4, and MPO expression in CNS tissues of EAE mice. **(A–D)** Western blot analysis of RGMa, BMP4, and MPO protein levels in brain tissue. **(A)** Representative blots of RGMa, BMP4, and MPO. **(B–D)** Densitometric quantification of RGMa **(B)**, BMP4 **(C)**, and MPO **(D)** protein expression. RGMa-mAb treatment significantly reduced protein levels compared with EAE mice. **(E–H)** Western blot analysis of RGMa, BMP4, and MPO protein levels in spinal cord tissue. **(E)** Representative blots. **(F–H)** Densitometric quantification of RGMa **(F)**, BMP4 **(G)**, and MPO **(H)** protein expression. RGMa-mAb treatment significantly decreased protein expression. **(I-N)** RT-qPCR quantification of RGMa, BMP4, and MPO mRNA levels in brain **(I–K)** and spinal cord **(L–N)** tissues. Data are presented as mean ± SD from three independent biological experiments (n = 3 per group). Statistical significance was determined by one-way ANOVA followed by multiple-comparison testing. *p < 0.05 and **p < 0.01 versus Control group; #p < 0.05 and ##p < 0.01 versus EAE group. BMP4, bone morphogenetic protein 4; EAE, experimental autoimmune encephalomyelitis; RGMa, repulsive guidance molecule alpha; RGMa-mAb, repulsive guidance molecule-A monoclonal antibody; RT-qPCR, reverse transcription polymerase chain reaction.

Preliminary analyses suggested that RGMa and BMP4 expression increased during EAE progression. To further examine their association with neutrophil activation, we measured protein and mRNA levels of RGMa, BMP4, and MPO in the brain and spinal cord of EAE mice. Western blot analysis revealed elevated expression of RGMa, BMP4 and MPO in EAE mice compared with controls (*p* < 0.05 for all comparisons), while RGMa-mAb treatment was associated with a reduction in the expression of these proteins (*p* < 0.05 for all comparisons; **Figures 5A–H**). Consistently, quantitative PCR analysis revealed that mRNA expression levels of *Rgma*, *Bmp4*, and *Mpo* were markedly increased in EAE mice (*p* < 0.05 for all comparisons), and RGMa-mAb intervention effectively suppressed the expression of these genes (*p* < 0.05 for all comparisons; **Figures 5I–N**). These results suggest that RGMa is linked to neutrophil-associated BMP4-SMAD signaling during neuroinflammation and that RGMa-mAb treatment can modulate this signaling axis.

**Figure 6:**
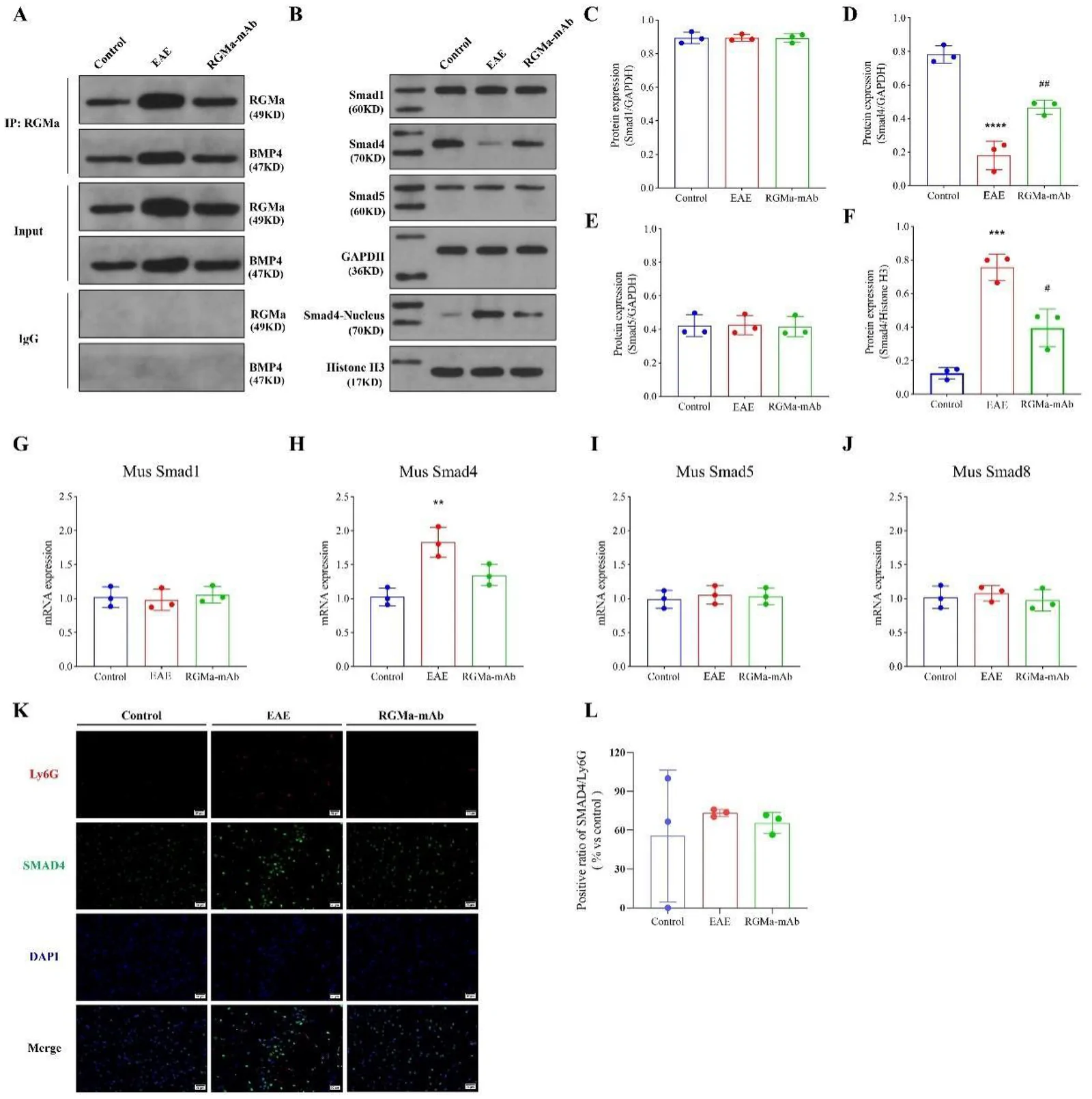
RGMa-associated alterations in BMP4-SMAD signaling in neutrophils of EAE Mice. **(A)** Co-immunoprecipitation (co-IP) validation of RGMa-BMP4 interaction in brain tissue. Input and IP lanes are shown; IP was performed using anti-RGMa antibody. RGMa-mAb treatment reduced expression of both proteins. **(B–F)** Western blot analysis of Smad family proteins in brain tissue. **(B)** Representative WB images for Smad1, Smad4 (cytoplasmic and nuclear), and Smad5. **(C–E)** Densitometric quantification of Smad1 **(C)**, Smad5 **(E)**, and cytoplasmic Smad4 **(D)**. **(F)** Quantification of nuclear Smad4. RGMa-mAb treatment significantly suppressed nuclear Smad4 accumulation compared with EAE mice. **(G–J)** RT-qPCR analysis of Smad1, Smad4, Smad5, and Smad8 mRNA levels in brain tissue. **(K, L)** Immunofluorescence detection of Smad4 and Ly6G in brain tissue. Ly6G+ neutrophils (red), Smad4 (green), nuclei counterstained with DAPI (blue). Representative images **(K)** and quantification of the proportion of Smad4^+^ neutrophils relative to total Ly6G^+^ cells **(L)** are shown. EAE mice exhibited an increased proportion of Smad4^+^ neutrophils compared with controls, whereas RGMa-mAb treatment showed a trend toward reduction and partial normalization. Scale bars = 20 μm. Data represent mean ± SD from three independent biological experiments (n = 3 per group). Quantitative immunofluorescence analyses were performed using three randomly selected microscopic fields per tissue section. Statistical analyses were performed using one-way ANOVA followed by multiple-comparison testing. *p < 0.05 and **p < 0.01 versus Control group; #p < 0.05 and ##p < 0.01 versus EAE group. BMP4, bone morphogenetic protein 4; DAPI, 4’,6-diamidino-2-phenylindole; EAE, experimental autoimmune encephalomyelitis; RGMa-mAb, repulsive guidance molecule-A monoclonal antibody; Smad, mothers against decapentaplegic homolog; Ly6G, lymphocyte antigen 6 complex locus G.

To further investigate the mechanistic link between RGMa and BMP4 signaling in the CNS, we performed co-immunoprecipitation (co-IP) on brain tissue lysates. As shown in **Figure 6A**, RGMa and BMP4 were detected in the input lanes, and BMP4 was also present in the RGMa immunoprecipitate, confirming a physical interaction between RGMa and BMP4 in EAE mice. Treatment with RGMa-mAb reduced the expression of both proteins, consistent with pathway inhibition.

We next examined the downstream Smad signaling. Western blot analysis showed no significant differences in Smad1 or Smad5 protein levels among the groups (**Figures 6B–F**). In contrast, Smad4 exhibited pronounced nuclear translocation in EAE mice, which was significantly suppressed by RGMa-mAb treatment (*p* = 0.0171). RT-qPCR analysis corroborated these findings, showing reduced Smad4 mRNA expression following RGMa-mAb intervention (**Figures 6G–J**).

To assess whether this pathway influences neutrophil activation, we performed immunofluorescence co-staining for Smad4 and the neutrophil marker Ly6G. Rather than quantifying only double-positive cells, we calculated the proportion of Smad4-positive neutrophils relative to the total neutrophil population (Ly6G^+^ cells) in brain tissue to minimize potential artifacts from non-specific staining or nuclear overlap. EAE mice exhibited an increased proportion of Smad4^+^ neutrophils compared with controls, whereas RGMa-mAb treatment showed a trend toward reduction and partial normalization toward control levels (**Figures 6K, L**).

**Figure 7:**
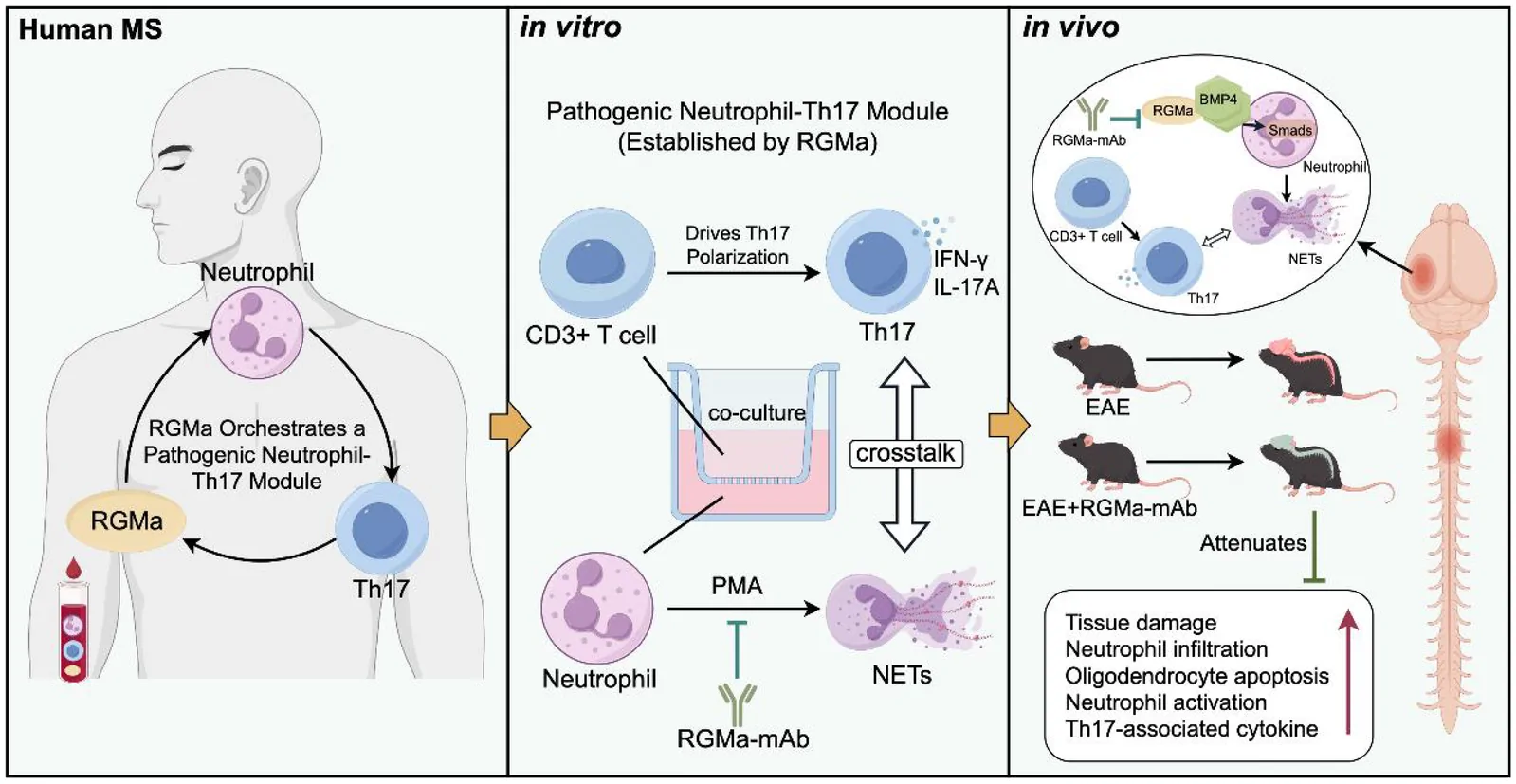
Proposed working model of RGMa-associated neutrophil-Th17 inflammatory interactions in CNS autoimmunity. Schematic summary of mechanistic insights from this study. RGMa may be associated with BMP4-SMAD-related signaling alterations, accompanied by neutrophil-associated inflammatory activation and the release of inflammatory mediators (e.g., MPO and NE). Activated neutrophils may subsequently facilitate Th17 cell differentiation and cytokine production (e.g., IL-17A and IFN-γ), potentially contributing to amplification of neuroinflammatory responses. RGMa-mAb treatment was associated with attenuation of BMP4-SMAD-related signaling changes, neutrophil-associated inflammatory responses, Th17-associated cytokine production, and CNS pathological changes *in vivo*. The model integrates evidence from clinical samples, co-culture experiments, and EAE studies.

These observations, together with the accompanying changes in BMP4-SMAD-related signaling, support an association between RGMa signaling and neutrophil-associated inflammatory activation during CNS inflammation. Based on these findings, we propose a working model in which RGMa-associated inflammatory responses may involve BMP4-SMAD-related signaling alterations and contribute to neutrophil-associated Th17 inflammatory responses in CNS autoimmunity (**Figure 7**).

## 4 Discussion

In the present study, we investigated the role of RGMa in neutrophil-associated Th17 inflammatory responses in CNS autoimmunity. Building upon previous studies that primarily focused on T-cell-associated RGMa signaling, our findings extend RGMa-associated neuroinflammatory regulation toward neutrophil-Th17 immune interactions. Using clinical samples, *in vitro* co-culture systems, and the EAE model, we found that RGMa inhibition attenuated neutrophil- and Th17-associated inflammatory responses and was accompanied by modulation of BMP4-SMAD-related signaling. These findings support the involvement of RGMa in neutrophil-associated neuroinflammatory regulation and provide a foundation for further mechanistic investigation.

Clinically, the relevance of this inflammatory module is reflected in the peripheral immune profile of MS patients: during the acute phase, RGMa levels were positively associated with the neutrophil activation marker NE and the Th17 effector cytokine IL-17A, whereas these relationships were no longer observed during remission. This dynamic pattern suggests that RGMa-associated neutrophil-Th17 inflammatory interactions may participate in active neuroinflammatory responses rather than representing a secondary bystander phenomenon.

Consistent with these clinical observations, *in vitro* co-culture experiments showed that RGMa blockade attenuated the ability of activated neutrophils to promote Th17 differentiation and cytokine production and was also associated with reduced T-cell chemotactic responses. Together, these findings support the concept that RGMa may contribute to innate-adaptive immune interactions involved in CNS autoimmunity.

The involvement of this inflammatory axis was further supported by *in vivo* studies in the EAE model. RGMa-mAb treatment ameliorated neurological deficits and was associated with reduced MPO and IL-17A expression in CNS tissues. In addition, RGMa inhibition reduced the spatial association between neutrophil- and Th17-associated inflammatory markers within CNS lesions, accompanied by attenuation of inflammatory pathology. Together, these findings suggest that modulation of neutrophil-Th17 inflammatory interactions may contribute to the reduction of neuroinflammatory responses in EAE.

In contrast to previous studies focusing on RGMa’s downstream regulation of T cell adhesion and migration via Rap1-ICAM-1 signaling (19), our findings extend RGMa-associated immune regulation toward neutrophil-associated inflammatory responses and their relationship with Th17-associated immunity. These observations support the possibility that RGMa may participate in broader innate-adaptive immune interactions in CNS autoimmunity and further suggest its potential relevance as a therapeutic target in neuroinflammatory disease.

At the mechanistic level, neutrophils are recognized to promote pathogenic Th17 cell differentiation through multiple pathways, including (i) the secretion of polarizing cytokines such as IL-1β and IL-23; and (ii) the release of NETs and granular proteins (24, 25). Emerging evidence indicates that NET components (24, 26) can contribute to Th17 polarization, for instance, through Toll-like receptor 2 (TLR2) (24) pathways. Our data support an association between RGMa-BMP4-SMAD signaling and neutrophil-associated inflammatory regulation. We observed that RGMa physically interacts with BMP4 in inflamed CNS tissue, accompanied by alterations in SMAD-related signaling and SMAD4 nuclear translocation, which may contribute to changes in neutrophil activation states (27) and potentially facilitate NET-related responses. In addition, the increased neutrophil populations observed following PMA stimulation may reflect activation-associated cellular changes and potentially altered neutrophil survival states, although these mechanisms were not specifically distinguished in the present study. This mechanistic insight unifies prior observations linking RGMa-BMP4 signaling to vascular and T cell modulation (22, 28) while revealing its broader influence on innate immune programming. These observations are also consistent with previous studies showing that modulation of intracellular inflammatory signaling pathways, including MAPK-, STAT3-, and cathepsin-associated signaling, can influence Th1/Th17-related immune responses and ameliorate EAE pathology (29–32).

In our study, we focused primarily on the BMP4-SMAD pathway based on our previous findings implicating this signaling cascade in neutrophil-associated inflammatory responses (27). While RGMa has also been linked to neogenin-associated signaling involved in T-cell regulation and axonal guidance (19, 33), a comprehensive dissection of all downstream RGMa signaling pathways was beyond the scope of the current work. An important unresolved question is whether RGMa acts directly on neutrophils through a specific receptor (e.g., neogenin or another unidentified receptor) or indirectly via secondary mediators. Our transwell co-culture data support a model in which RGMa primarily modulates the neutrophil secretome, which subsequently influences Th17-associated inflammatory responses. Although the present study evaluated RGMa-related signaling changes at both the protein and mRNA levels, the precise receptor-mediated mechanisms, the relationship between RGMa and BMP4-SMAD signaling, and the downstream transcriptional programs linking these pathways to neutrophil-Th17-associated inflammation remain incompletely understood. In particular, whether RGMa acts directly through neutrophil-specific receptors or indirectly via secondary mediators requires further investigation. Future studies using receptor-blocking approaches or neutrophil-specific genetic models will be important to further dissect these mechanisms.

Our findings support a potential role for RGMa in neutrophil-Th17-associated inflammatory responses in CNS autoimmunity. Together with recent insights from peripheral inflammatory diseases, these data suggest that RGMa-related signaling may participate in upstream regulation of neutrophil-Th17 inflammatory circuits. The established paradigm, supported by studies such as Han et al. (24, 34), describes the IL-23/Th17 loop as a self-amplifying circuit wherein Th17-derived IL-17 recruits and activates neutrophils, which in turn reinforce Th17 responses via cytokines such as IL-1β and IL-23. Our data suggest that RGMa inhibition is associated with attenuation of both neutrophil- and Th17-associated inflammatory responses, accompanied by alterations in BMP4-SMAD-related signaling. In parallel, Han et al. identified CD39^+^CD9^+^ interstitial macrophages as a suppressive mechanism limiting neutrophil NETosis downstream (34). Collectively, these findings support the concept that multiple regulatory mechanisms may converge on neutrophil-associated inflammatory networks in CNS autoimmunity.

Our findings also suggest a potential therapeutic relevance of RGMa-associated immune regulation in CNS autoimmunity. However, this potential therapeutic effect is still at the preclinical stage, and considerable work remains before clinical application can be realized. Current T cell-centric strategies in MS have shown variable efficacy (35), implying the existence of upstream microenvironmental drivers of pathogenic immunity. Converging evidence from the current study and prior work in NMO (36) raises the possibility that modulation of RGMa-associated myeloid-lymphoid immune interactions may represent an additional strategy for attenuating neuroinflammatory responses. Rather than directly targeting effector cells, such approaches may influence broader inflammatory communication networks involved in CNS autoimmunity. Consistent with this concept, early clinical data on the recombinant anti-RGMa antibody Elezanumab have already demonstrated neuroprotective potential (37), while our work provides the preliminary mechanistic evidence supporting its immunomodulatory efficacy.

Despite the robust mechanistic insights provided by the present study, several limitations warrant consideration. First, the clinical sample size was relatively modest, which limits the statistical power and generalizability of the findings. Broader translational validation in larger patient cohorts will be essential to confirm the observed associations. Second, while our study focused on neutrophil-associated Th17 responses, RGMa may influence additional immune cell populations and pathways, which remain to be fully explored. Third, methodological limitations, including the potential confounding effects of DMTs on peripheral immune biomarkers during remission, the use of a single internal reference gene for RT-qPCR, absence of viability dye staining and protein transport inhibitors in flow cytometry, and the lack of additional antibody or genetic controls, highlight areas where experimental rigor could be enhanced. Collectively, these considerations underscore the need for future studies employing expanded pathway-perturbation approaches, such as receptor blocking, BMP pathway inhibition, genetic knockdown/knockout models, and neutrophil-specific genetic models, as well as refined immunophenotyping (e.g., using oligodendrocyte lineage markers and neutrophil-associated markers), in larger cohorts, to validate and extend these findings and further define RGMa-mediated neuroinflammatory mechanisms.

In summary, our exploratory findings suggest that RGMa is associated with neutrophil-Th17 inflammatory responses in CNS autoimmunity and may contribute to neuroinflammatory regulation, potentially involving BMP4-SMAD-related signaling. RGMa-mAb treatment was associated with attenuation of neutrophil- and Th17-associated inflammatory responses in both *in vitro* and EAE models. These findings provide preliminary mechanistic insight into RGMa-associated neuroinflammatory signaling and support further investigation of RGMa-targeted therapeutic strategies in neuroinflammatory diseases.

## Data Availability

The raw data supporting the conclusions of this article will be made available by the authors, without undue reservation.
