# Supplementary material for: Repulsive guidance molecule-a regulates neutrophil-associated Th17 responses in CNS autoimmunity
Source: Front Immunol. 2026 Aug 19;17:1941039. doi: 10.3389/fimmu.2026.1941039 (PMC13533730; doi:10.3389/fimmu.2026.1941039)
Supplement: Supplementary file 1 [file DataSheet1.docx]

Supplementary Figure S1. Representative flow cytometry gating strategies for Th17 cells and neutrophils.

(A) Th17-cell gating. CD4⁺IL-17A⁺ T cells were identified from co-cultured lymphocytes. Cells were first distinguished from debris and dead cells using forward scatter (FSC) and side scatter (SSC), and doublets were excluded using FSC-H/FSC-W gating. A 2D fluorescence plot (CD4 vs IL-17A) was used to define quadrants based on isotype and unstained controls, with the percentage of Th17 cells calculated from the upper-right quadrant.

(B) Neutrophil gating. CD11b⁺Ly-6G/Ly-6C⁺ neutrophils were identified from co-cultured myeloid cells using the same initial gating strategy to exclude debris and doublets. Quadrants were defined based on negative controls, and the percentage of neutrophils was calculated from the CD11b⁺Ly-6G/Ly-6C⁺ population in the upper-right quadrant.

Data are representative of three independent biological experiments (n = 3).

**Supplementary Table 1. Reagents and Antibody Information**

| **Reagent** | **Manufacturer** | **Article Number** | — | — | — |
| --- | --- | --- | --- | --- | --- |
| Unasnemab (RGMa monoclonal antibody; RGMa-mAb） | MedChemExpress | HY-P99959 | — | — | — |
| PMA | MedChemExpress | HY-18739 | — | — | — |
| PTX | MedChemExpress | HY-112779 | — | — | — |
| MOG35-55 | GL Biochem, Shanghai | 51716 | — | — | — |
| CFA | Sigma-Aldrich | F5881 | — | — | — |
| Mycobacterium tuberculosis H37Ra | BD | 231141 | — | — | — |
| Complete medium for peripheral blood neutrophils of humans | Procell | CM-H197 | — | — | — |
| Human Peripheral Blood Lymphocyte Isolation Solution (Prepared By Ficoll) | Beijing Solarbio Biotechnology Co., Ltd. | P8900 | — | — | — |
| Crystal violet staining solution | Beyotime Biotechnology | C0121 | — | — | — |
| HiScript III 1st Strand cDNA Synthesis Kit | Nanjing Novaz Bio-Technology Co., Ltd. | R-312-01 | — | — | — |
| FBS | Procell | 164210 | — | — | — |
| **Antibody/Target protein (expected MW, kDa)** | **Manufacturer** | **Article Number** | **Host/Type** | **Concentration/Dilution Ratio** | **Usage** |
| RGMa antibody (~49 kDa) | Proteintech | 12387-1-AP | Rabbit/Polyclonal | 1:1000 | WB |
| BMP4 antibody (~47 kDa) | Proteintech | 12492-1-AP | Rabbit/Polyclonal | 1:1000 | WB |
| Neutrophil Elastase antibody | Beijing Bioss Biotechnology Co., Ltd. | bs-23549R | Rabbit/Polyclonal | 1:100 | IF |
|  |  |  |  | 1:1000 |  |
| MPO antibody (~59 kDa) | Beijing Bioss Biotechnology Co., Ltd. | bs-4943R | Rabbit/Polyclonal | 1:100 | IF |
|  |  |  |  | 1:1000 | WB |
| IL-17A antibody | Proteintech | 66148-1-Ig | Mouse/Monoclonal | 1:200 | IF |
| RORγt antibody | Santa Cruz Biotechnology | sc-365476 | Mouse/Monoclonal | 1:200 | IF |
| MOG antibody | Santa Cruz Biotechnology | sc-166172 | Mouse/Monoclonal | 1:200 | IF |
| SMAD1 antibody (~60 kDa) | Cell Signaling Technology | 6944T | Rabbit/Monoclonal | 1:1000 | WB |
| SMAD4 antibody (~70 kDa) | Cell Signaling Technology | 38454T | Rabbit/Monoclonal | 1:1000 | WB |
| SMAD5 antibody (~60 kDa) | Cell Signaling Technology | 12534T | Rabbit/Monoclonal | 1:1000 | WB |
| GAPDH antibody (~36 kDa) | Proteintech | 10494-1-AP | Rabbit/Polyclonal | 1:2000 | WB |
| Histone H3 antibody (~17 kDa) | Proteintech | 17168-1-AP | Rabbit/Polyclonal | 1:1000 | WB |
| Goat anti-Rabbit IgG H&L | Beijing Bioss Biotechnology Co., Ltd. | bs-0295G | Goat/Rabbit | 1:10000 | WB secondary antibody |
| Goat anti-Mouse IgG H&L | Beijing Bioss Biotechnology Co., Ltd. | bs-0296G | Goat/Mouse | 1:10000 | WB secondary antibody |
| CD11b Monoclonal Antibody (M1/70), PE, eBioscience™ | Thermo Fisher Scientific | 12-0112-81 | Rat/Monoclonal | 0.125 µg/test | Flow |
| Ly-6G/Ly-6C Monoclonal Antibody (RB6-8C5), FITC, eBioscience™ | Thermo Fisher Scientific | 11-5931-81 | Rat/Monoclonal | 0.125 µg/test | Flow |
| CD4 Monoclonal Antibody (GK1.5), FITC, eBioscience™ | Thermo Fisher Scientific | 11-0041-81 | Rat/Monoclonal | 0.25 µg/test | Flow |
| IL-17A Monoclonal Antibody (eBio17B7), PE, eBioscience™ | Thermo Fisher Scientific | 12-7177-81 | Rat/Monoclonal | 0.125 µg/test | Flow |
| **Enzyme-linked immunosorbent assay kit** | | | | | |
| **Reagent** | **Manufacturer** | **Article Number** | **species reactivity** | **specifications** | — |
| Human IL-17A Enzyme-Linked Immunosorbent Assay Kit | Quanzhou Ruixin Biotechnology Co., Ltd. | RX106160H | Human sample | 96T | — |
| Human IFN-γ Enzyme-Linked Immunosorbent Assay Kit | Quanzhou Ruixin Biotechnology Co., Ltd. | RX106205H | Human sample | 96T | — |
| Human NE Enzyme-Linked Immunosorbent Assay Kit | Quanzhou Ruixin Biotechnology Co., Ltd. | RX104801H | Human sample | 96T | — |
| Human CD66 Enzyme-Linked Immunosorbent Assay Kit | Quanzhou Ruixin Biotechnology Co., Ltd. | RX103277H | Human sample | 96T | — |
| Human BMP4 Enzyme-Linked Immunosorbent Assay Kit | Quanzhou Ruixin Biotechnology Co., Ltd. | RX105865H | Human sample | 96T | — |
| Human RGMa Enzyme-Linked Immunosorbent Assay Kit | Quanzhou Ruixin Biotechnology Co., Ltd. | RXM2D187406 | Human sample | 96T | — |
| Human Ly6G6D Enzyme-Linked Immunosorbent Assay Kit | Jiangsu Jingmei Biotechnology Co., Ltd. | JM-7499H1 | Human sample | 96T | — |
| Mouse CD66 Enzyme-Linked Immunosorbent Assay Kit | Quanzhou Ruixin Biotechnology Co., Ltd. | RX203087M | Mouse sample | 96T | — |
| Mouse IFN-γ Enzyme-Linked Immunosorbent Assay Kit | Quanzhou Ruixin Biotechnology Co., Ltd. | RX203097M | Mouse sample | 96T | — |
| Mouse IL-17 Enzyme-Linked Immunosorbent Assay Kit | Quanzhou Ruixin Biotechnology Co., Ltd. | RX203067M | Mouse sample | 96T | — |
| Mouse MPO Enzyme-Linked Immunosorbent Assay Kit | Quanzhou Ruixin Biotechnology Co., Ltd. | RX200824M | Mouse sample | 96T | — |

**Supplementary Table 2.** Mouse primers and sequences

| Primer name | Primer sequence (5′ to 3′) | | Product size (bp) |
| --- | --- | --- | --- |
| GAPDH | F | ATGGGTGTGAACCACGAGA | 229 |
|  | R | CAGGGATGATGTTCTGGGCA |  |
| RGMA | F | CTTACCACTCGGCTGTCCAT | 202 |
|  | R | AGGCCGCAGTGAGTGTAGTT |  |
| BMP4 | F | TGATACCTGAGACCGGGAAG | 190 |
|  | R | AGCCGGTAAAGATCCCTCAT |  |
| MPO | F | CACCCTCTTTGTTCGAGAGC | 154 |
|  | R | CAACACCAAGGGCAGGTAGT |  |
| SMAD 1 | F | AAATCAGCAGAGGAGATGTTCA | 164 |
|  | R | GCGGTTCTTATTGTTGGACGGA |  |
| SMAD 4 | F | CCATTTCCAATCATCCTGCT | 193 |
|  | R | TCAATCGCTTCTGTCCTGTG |  |
| SMAD 5 | F | CAACGCTCCTTTCCCCTTATCT | 272 |
|  | R | ACCAGTGTTTGGGCTCCTCATA |  |
| SMAD 8 | F | ACCGACCCTTCCAATAACAGGA | 128 |
|  | R | TCGCCCCCAACGTAGTACAAAT |  |

**Supplementary Table 3.** Human primers and sequences

| Primer name | Primer sequence (5′ to 3′) | | Product size (bp) |
| --- | --- | --- | --- |
| GAPDH | F | TCAAGAAGGTGGTGAAGCAGG | 115 |
|  | R | TCAAAGGTGGAGGAGTGGGT |  |
| RORγt | F | GAGGAAGTCCATGTGGGAGA | 167 |
|  | R | TCCTAACCAGCACCACTTCC |  |
| IL-17 | F | TGTGATCTGGGAGGCAAAGT | 168 |
|  | R | CCCACGGACACCAGTATCTT |  |
| IL-22 | F | ACTTCCAGCAGCCCTATATCACCAA | 104 |
|  | R | GAACAGTTTCTCCCCAATGAGACGA |  |
| INFγ | F | TTCAGCTCTGCATCGTTTTG | 246 |
|  | R | TCTTTTGGATGCTCTGGTCA |  |
